# Factors Affecting Health Related Quality of Life in Hospitalized Patients with Heart Failure

**DOI:** 10.1155/2017/4690458

**Published:** 2017-11-01

**Authors:** Georgia Audi, Aggeliki Korologou, Ioannis Koutelekos, Georgios Vasilopoulos, Kostas Karakostas, Kleanthi Makrygianaki, Maria Polikandrioti

**Affiliations:** ^1^Faculty of Health and Caring Professions, Department of Nursing, Technological Educational Institute of Athens, Athens, Greece; ^2^Thriasio General Hospital, Elefsina, Athens, Greece; ^3^Alexandra General Hospital, Athens, Greece

## Abstract

This study identified factors affecting health related quality of life (HRQOL) in 300 hospitalized patients with heart failure (HF). Data were collected by the completion of a questionnaire which included patients' characteristics and the Minnesota Living with Heart Failure Questionnaire (MLHFQ). Analysis of data showed that the median of the total score of MLHFQ was 46 and the median of the physical and mental state was 22 and 6, respectively. Also, participants who were householders or had “other” professions had lower score of 17 points and therefore better quality of life compared to patients who were civil/private employees (*p* < 0.001 and *p* < 0.001, resp.). Patients not receiving anxiolytics and antidepressants had lower quality of life scores of 6 and 15.5 points, respectively, compared to patients who received (*p* = 0.003 and *p* < 0.001, resp.). Patients with no prior hospitalization had lower score of 7 points compared to those with prior hospitalization (*p* = 0.002), whereas patients not retired due to the disease had higher score of 7 points (*p* = 0.034). Similar results were observed for the physical and mental state. Improvement of HF patients' quality of life should come to the forefront of clinical practice.

## 1. Introduction

Heart failure (HF) is a global public and clinical health problem that is expanding at an alarming rate due to the ageing of population and the improvement in diagnosis and treatment of cardiovascular disease [1–3].

More than 5.8 million in the United States and more than 23 million worldwide suffer from the disease [2, 3]. Each year, 550,000 new cases are diagnosed [2–4], while this number is predicted to reach 1.5 million annually by 2040 [4].

Disease prevalence is increasing with age; in more detail, it affects approximately 2–5% of adults aged 65–75 and >10% of adults aged 80 and older [5, 6]. HF is more frequent in men than in women [6].

This life-threatening disease is also associated with high morbidity, mortality, and rehospitalizations [2, 3]. After diagnosis of HF, survival estimates are 50% and 10% at 5 and 10 years, respectively [2]. In regard to severe HF, more than 50% of patients die within one year after diagnosis [2, 5]. Moreover, HF is responsible for 1 million hospitalizations, in the United States and Europe annually [7, 8]. After discharge, rates of rehospitalization approach 30% within 60 to 90 days [7]. Exacerbation of symptoms accounts for 50% of readmissions within six months [4].

Although HF may be caused by several medical states, the prevalent etiology is ischemic heart disease mainly in the western world. In more detail, approximately 36% of patients with myocardial infarction will develop heart failure after 7-8years [9, 10].

HF imposes a tremendous burden on patients, care givers, society, and health care system of each country. Indeed, HF patients experience various physical and psychosocial problems that affect their health related quality of life (HRQOL) [1, 11]. HF patients' HRQOL is a valuable measure for the outcome of the disease [11].

To the best of our knowledge, little is known about determinants of HRQOL in the hospitalized HF population in Greece.

*The aim* of this study was to identify factors affecting health related quality of life (HRQOL) in hospitalized HF patients.

## 2. Methods

### 2.1. Study Population and Design

The sample of the study consisted of 300 hospitalized HF patients (167 men and 133 women) in cardiology clinics of 4 public hospitals in Attica between June 2015 and October 2015.

This patient sample was a convenience one (convenience sample).

Inclusion criteria were (a) an established diagnosis of HF, (b) at least 2 days' hospitalization in cardiology clinics because of HF, and (c) sufficient understanding of the Greek language. Participation was anonymous and voluntary; however, patients were able to withdraw from the study at any moment.

Patients were excluded if they had a concurrent diagnosis of other life-threatening diseases (e.g., cancer) or a chronic severe psychiatric condition (e.g., psychosis), had a history of alcohol abuse in the past six months, or were unable to communicate with the researcher or to give their written consent.

The process of filling out the questionnaires took between 15 and 30 minutes.

*Ethical Considerations*. All participants gave their written informed consent. The study was approved by the Ethics Committee for Medical Research in each participating hospital and was conducted in accordance with the Declaration of Helsinki (1989) of the World Medical Association.

Data collection was performed by the method of the interview using a questionnaire developed by the researchers of the study so as to fully serve its purposes.

The data collected for each patient included sociodemographic characteristics (e.g., gender, age, education level, job, and residency) and characteristics concerning the state of health: (a) clinical characteristics (e.g., medication, other diseases, years of having the problem, prior hospitalization, frequency, and days of hospitalization) and (b) other self-report characteristics (e.g., level of information about the state of health, relations with the medical and nursing staff, considering themselves as anxious, and retirement or absence from work due to the cardiac problem).

### 2.2. Health Related Quality of Life (HRQOL) in HF Patients

The “Minnesota Living with Heart Failure” questionnaire (MLHFQ) was used to evaluate the health related quality of life (HRQOL) in hospitalized HF patients. It is the most widely known and used disease-specific instrument and has been translated in at least 34 languages with proven reliability and validity [11, 12].

This scale which was proposed in 1986 by the University of Minnesota [12] consists of 21 questions asking about how much the disease and its treatment had affected the patient's life in the last month (4 weeks). Respondents are able to answer each question in a Likert type scale (scores from 0: no effect to 5: very much).

The Minnesota Living with Heart Failure Questionnaire (MLHFQ) measures the total score of HRQOL and two separate dimensions of HRQOL: (a) the physical state of the patient and (b) the mental state of the patient. In more detail, from MLHFQ, three scores were calculated: (a) the physical state of the patients (range: 0–40), (b) the mental state of patients (range: 0–25), and (c) the total score of quality of life from all 21 questions (range: 0–105).

The score assigned to the questions is summed separately to questions that assess physical state, for those that assess mental state and all 21 questions together to an aggregate score, the total quality of life.

Higher values of scores indicate poorer quality of life.

### 2.3. Statistical Analysis

Categorical data are presented in absolute and relative (%) frequencies, whereas continuous data are presented with a median (interquartile range) if normality is not followed (the criterion was tested with Kolmogorov-Smirnov). The Kruskal-Wallis test was used to test the existence of association between the quality of life and a factor with more than two categories, while the Mann–Whitney test for the existence of association between the quality of life and a factor with two categories. Moreover, multiple linear regression was performed to conclude which independent statistically significant factors affect the quality of life. Results are presented as regression coefficients b (b-coefficients) and 95% confidence interval (95% CI). The statistically significant level of 5% was observed. All statistical analyzes were performed using the SPSS version 20 package (SPSS Inc., Chicago, Il, USA).

## 3. Results

### 3.1. Descriptive Results

In total, 300 hospitalized HF patients were enrolled in the study, of whom men constitute 55.7%, while 90% of the sample was aged over 60 years.

37.3% had a high school education, while 65% were pensioners. The majority of patients were leaving in Attica (43.7%) (Table 1).

28.7% and 4.7% of participants were receiving medication with anxiolytics and antidepressants, respectively.

In 42.7%, other diseases coexisted. Only one person had not been informed at all about the health problem. The majority of patients (34.7%) had the disease for 6–10 years, while 55% were hospitalized once a year because of the problem.

66.7% of the participants characterized themselves as anxious and the vast majority of the patients reported that they had good or very good relations with both the medical and nursing staff.

The median duration of hospitalization in the clinic was 5 days (Table 2).

Table 3 presents the results relating to participants' quality of life. It is observed that at least 50% of the patients had scores below 46 (median) *n* in the total score of quality of life and 22 and 6 in physical and mental score, respectively. These values indicate moderate effects of heart failure in the quality of life of patients.

### 3.2. Association between Quality of Life and Patients' Characteristics

Tables 4 and 5 present the associations between quality of life and patients' characteristics.

The total score for quality of life was statistically significantly associated with educational level (*p* = 0.002), job (*p* < 0.001), and place of residence (*p* < 0.001) (Table 4). More specific, patients with a high school education level (median 54) scored higher meaning that they had a worse quality of life in relation to the remaining patients. Also, employees (civil/private employees) had a worse quality of life (median 58.5). Finally, patients who resided in a county capital had higher scores (worse quality of life, median 54) compared to patients living in Attica or a small town.

Physical state was statistically significantly associated with educational level (*p* = 0.003) and job (*p* = 0.007). Specifically, patients with secondary educational level (median 25) and employees (civil/private employees) (median 26) scored higher meaning that they were in worse physical state in relation to the remaining patients.

Mental state was statistically significantly associated with age (*p* < 0.001), educational level (*p* < 0.001), job (*p* < 0.001), and place of residence (*p* < 0.001). Patients aged under 60 years had higher scores of mental state (worse mental state, median 11) compared to patients older than 60. Patients with high school educational level scored higher meaning that they were in worse mental state (median 12) compared to patients with a lower level of education. Similarly for patients who were employees (civil/private employees) and those who were living in a county capital, they were also in worse mental state (median 12 and 11, resp.).

Table 5 presents the association between quality of life and patients' characteristics (clinical and self-report characteristics).

The total score of patients' quality of life was statistically significantly associated with the following: the medication with anxiolytics (*p* < 0.001) and antidepressants (*p* < 0.001), years of having the problem (*p* < 0.001), if they had been hospitalized before (*p* = 0.005), if they considered themselves anxious (*p* < 0.001), and if they were retired or absent from work because of the problem (*p* < 0.001 and *p* < 0.001, resp.). More specifically, it was found that patients taking antidepressants and anxiolytics had a worse quality of life (median 54 and 64, resp.); patients suffering from 6 to 10 years had also worse quality of life (median 54) and similarly the ones that had been hospitalized before (median 46) and those who had not retired or were not absent from work because of the problem (median 46 and 46, resp.).

Physical state was statistically significantly associated with the following: the medication with anxiolytics (*p* < 0.001) and antidepressants (*p* < 0.001), whether suffering from another disease (*p* < 0.001), the degree of information (*p* = 0.031), years of having the problem (*p* < 0.001), if the patient had been hospitalized before (*p* = 0,001), the frequency of hospitalization (*p* = 0.035), if patients considered themselves anxious (*p* < 0.001), and if they were retired or absent from work because of the problem (*p* = 0.003 and *p* = 0.023, resp.).

Specifically, patients who were taking anxiolytics and antidepressants, were suffering from another disease, were “a little” aware of their problem, were suffering the disease for 6–10 years, were hospitalized before, were considering themselves anxious, and had not been retired or absent from work because of the problem were in a worse physical state than other patients.

Mental state was statistically significantly associated with the following: the medication with anxiolytics (*p* < 0.001) and antidepressants (*p* < 0.001), years of having the problem (*p* < 0.001), if a relative suffered from heart problems (*p* = 0.001), hospitalization frequency (*p* = 0.015), if patients considered themselves anxious (*p* < 0.001), if they were retired or absent from work because of the problem (*p* = 0.002 and *p* = 0.001, resp.), and the relations with the medical staff (*p* = 0.015) and the nursing staff (*p* < 0.001). Specifically, patients who were taking anxiolytics and antidepressants, were suffering for 6–10 years from the problem, were considering themselves anxious, and had not retired or were absent from work because of the problem and those who reported having substandard relations with the medical and nursing staff were in a worse mental state than other patients.

### 3.3. Assessing the Effect of the Factors on Quality of Life

Multivariate linear regression was performed to check which independent factors affect the quality of life of HF patients. As independent variables in the model were entered, the factors that were statistically significant associated with the quality of life in the univariate analysis.

From Table 6, it is concluded that patients who were householders or had “other” professions had lower score of 17 points and therefore better quality of life in relation to patients who were civil/private employees (*p* < 0.001 and *p* < 0.001, resp.). Patients who did not take anxiolytics and antidepressants had lower quality of life scores of 6 and 15.5 points, respectively, compared to patients who did (*p* = 0.003 and *p* < 0.001, resp.). In addition, patients who had not been hospitalized before had lower score of 7 points compared to patients that had been hospitalized before (*p* = 0.002), whereas patients that were not retired due to the problem had higher score of 7 points (*p* = 0.034).

From Table 7 we conclude that patients who were householders, were pensioners, or had “other” professions had lower scores of 6.4, 4.7, and 9.2 points, respectively, and hence better levels of physical state in relation to the patients who were civil/private employees (*p* < 0.001, *p* = 0.001, and *p* < 0.001, resp.). Patients who did not take anxiolytics had lower score of physical state of 2.8 points than patients who did (*p* = 0.005). Patients who did not have other diseases had better physical state score of 3.6 points (*p* < 0.001). Patients who were enough or a little informed about their state of health had worse physical state score of 2.3 and 8.7 points, respectively, than those who were very informed (*p* = 0.011 and *p* < 0.001, resp.). Patients suffering for 6 to 10 years and 11–15 years from the problem had worse physical state score of 4.4 and 5 points, respectively, than those who suffered for less than a year (*p* = 0.010 and *p* = 0.005, resp.). In addition, patients who had not been hospitalized before had better physical state score of 5 points (*p* = 0.001), whereas patients that were hospitalized more than three times a year had worse physical state score of 6 points in relation to those who were hospitalized once a year (*p* < 0.001). Finally, patients who had not retired because of the problem had worse physical state score of 3.8 points (*p* = 0.024).

Regarding mental state, patients aged 60–69 years had lower score of 1.9 points and therefore better mental state than patients aged <60 years (*p* = 0.024). Patients with secondary educational level had worse mental state score of 1.2 points than patients with primary educational level (*p* = 0.042). Patients who were householders, were pensioners, or had “other” professions had lower scores 6, 3, and 4.7 points, respectively, and hence better mental state in relation to patients who were civil/private employees (*p* < 0.001, *p* = 0.002, and *p* < 0.001, resp.). Patients living in a county capital had worse mental state score of 2.15 points than patients who lived in Attica (*p* = 0.008). Patients who did not take anxiolytics and antidepressants have lower mental state scores of 1.4 and 5.3 points, respectively, than patients who did (*p* = 0.005 and *p* < 0.001, resp.). Patients suffering for 2–5 years and 6–10 years from the problem had worse mental state score of 2.3 and 2.7 points, respectively, than those suffering for less than a year (*p* = 0.008 and *p* = 0.002, resp.). In addition, patients who were hospitalized more than 3 times a year had worse mental state score of 1.7 points than those who were hospitalized once a year (*p* = 0.005). Patients who characterized themselves anxious had better mental state score of 1.15 points. Finally, patients who had good or below moderate relationship with the medical staff had worse mental state score of 1.8 and 5 points, respectively, than patients who had very good relationship (*p* = 0.013 and *p* < 0.001, resp.).

## 4. Discussion

The results of the present study showed that patients who had high school education level, were employees (civil/private employees), and were residents in a county capital had worse quality of life.

Education level and its close association with socioeconomic status are predictive of reduced quality of life [4, 13]. A possible explanation is that low financial sources along with inability to understand medical instructions imply lack of adherence to treatment and, therefore, reduced effectiveness of disease management.

The finding of the present study that living in county capital was associated with reduced total quality of life is possibly attributed to the stressful everyday living. However, place of residency needs further scrutiny as it seems to influence quality of life, indirectly. For example, rural patients may have limited access to health care services including cardiac rehabilitation interventions and are more likely to be readmitted due to the exacerbations of disease [4].

Regarding age, the results revealed that HF patients aged under 60 years had worse mental state. Based on the knowledge that HF incidence increases with age, researchers would anticipate that older patients who experience several limitations such as cognitive impairment, loss of personal autonomy, or anxiety and depression may have poor quality of life. However, Erceg et al. [14] who also explored hospitalized patients found no correlation between age, gender, and quality of life. Moreover, the same researches showed the depressive symptoms, the higher NYHA class, the lower income, and the longer duration of heart failure as independent predictors of poor quality of life.

The finding that participants taking anxiolytics and antidepressants were in both physical and mental worse state is attributed to adverse outcomes of depression in HF patients' life. In more detail, depression involves physical impairment, limited social functioning, role restrictions or emotional distress, and high risk of hospitalization [15–18]. On the other end of the spectrum, HF patients need this medication to alleviate the emotional burden of this unpredictable disease and the shortened lifespan.

Also associated with both physical and mental worse state were the years of suffering from the disease (6–10 years) which may reflect symptoms' severity. HF patients often experience loss of functional independence in daily activities such as feeding, dressing, housekeeping, bathing, and walking [19]. The most common symptoms affecting quality of life are dyspnea at rest or on exertion, paroxysmal nocturnal dyspnea, orthopnea, and fatigue as well as lack of energy [20, 21].

It is noteworthy that evaluation is needed of all the changes that take place through years and that may exacerbate HF patients' quality of life such as inability to fulfill their prior role (social, professional, and family), diminished self-esteem, and distorted picture of themselves.

Data revealed that patients suffering from other diseases and those who had prior hospitalization were in a worse physical state. Comorbidities amongst HF patients, such as diabetes, peripheral vascular disease, and cerebrovascular disease, are responsible for poor performance in activities, for hospitalization, and for increased health care expenditures [20–23]. Unfortunately, in the present study, 42.7% suffered from another disease.

Interestingly, there is a high incidence of readmissions in HF patients with ≥50% of them to be readmitted to hospital within 6 months of discharge [24, 25]. Many reasons are held to be responsible for frequent rehospitalizations such as clinical and laboratory parameters, the overall disability, the inadequate self-monitoring, and treatment adherence failure [26–28]. Length of hospital stay is correlated inversely with overall quality of life [14]. In the present study, 82% of the participants had prior hospitalization due to the same reason.

Patients who considered themselves anxious were in a worse physical and mental state. Anxiety is characterized as a subjective unpleasant feeling emerging when anticipated events are experienced as a menace. Patients who characterize themselves as anxious usually perceive the disease and its inevitable consequences as a threat or as a loss of control on effects of the disease. Working individuals are socially active and do not easily accept the role of “patient.” On the other hand, some individuals when performing occupational tasks may feel lack of energy or diminished level of functional ability due to the disease which partially explains their worse physical and mental state [1, 29].

Also in a worse physical and mental state were patients who were absent or retired from work because of the problem. Theories about association between work and chronic illness are contradictory. On the one hand, individuals who are working are socially active and do not easily accept the role of “patient,” while, on the other hand, they may face difficulties during performing occupational tasks due to the lack of energy or the diminished level of functional ability [30]. Therefore, this finding raises concern about the appropriate time to retire from work.

Participants being “a little” aware of their health were in worse physical state. Patients with a knowledge deficit about the disease may underestimate its magnitude or fail to adhere to the therapeutic regimen [5]. Functional and cognitive limitations are the most common barriers in terms of disease knowledge, while only well-informed patients may take control for their own health [31, 32].

On the basis of these findings, we seek to determine the crucial role of health professionals to provide accurate information. Lack of awareness about the disease among HF patients and their families is not a rare issue since clinicians often (a) put more emphasis on therapy and have diminished available time for conversations with HF patients, (b) lack confidence to provide end life care, and (c) show reluctance to negotiate end-of-life issues or experience uncertainty whether patients wish to obtain an in-depth knowledge of the disease [33–36].

The key-factor in providing care of high quality to HF patients is constant assessment of their information needs which may vary in different stages of the disease. In more detail, elaborate information motivates individuals to seek help in the early stages of heart failure, whereas in advanced disease, patients' needs (physical, emotional, or practical) are frequently unmet as health professionals are unable to provide any further medical aid [37].

Clinical approaches will be most effective when tailored to patients' needs and preferences. Need for orientated approach is of fundamental importance, since, nowadays, it is widely acknowledged that the ultimate goal in HF treatment is not solely patients' survival but also improvement of their quality of life [38]. Other significant areas within the field of HF are to facilitate hospital to home transition by evaluating and meeting their needs, through multidisciplinary team approach including involvement of palliative care [39].

What is more intriguing is that data highlighted worse mental state in participants who reported having substandard relations with the medical and nursing staff. Poor communication between health professionals and patients is an obstacle to patients' effective self-care [40]. However, positive and therapeutic relations demand great effort. Though contact with a HF nurse is not associated with quality of life, it increases patients' satisfaction with treatment [41].

## 5. Study Limitations

The present study was cross-sectional and collected data at one point in time, thus not allowing for inferences or changes over time. A cross-sectional study does not allow the determination of a causal relation between quality of life and the sociodemographic and clinical variables.

The sampling method of the present study was a convenience one, which is not representative of HF patients in Greece, thus limiting the generalizability of the results. The strengths of the study include (a) the use of a wide spread instrument, (b) the number of HF patients, and (c) “hospitalization” as most research is conducted either in community or in outpatient department of hospital when they come for regular monitoring and follow-up.

## 6. Conclusions

Measuring health related quality of life is increasingly important in both clinical practice and research and constitutes a challenge for clinicians involved in the care of HF. Given the high incidence of comorbidities in heart failure, it is essential to provide multidisciplinary care involving other specialties apart from cardiologists.

Early assessment of factors affecting quality of life would have a positive effect on disease management and outcomes. The study findings underscore the importance of individualized care for HF patients and suggest future directions for research in this important area. Finally, clinicians should maintain focus on treating disease, maximizing life expectancy, and optimizing quality of life for HF patients at all disease stages.

## Figures and Tables

**Table 1:** Patients characteristics (*N* = 300).

	*N* (%)
*Sex*	
Male	167 (55.7%)
Female	133 (44.3%)
*Age*	
30–39	7 (2.3%)
40–49	7 (2.3%)
50–59	17 (5.7%)
60–69	98 (32.7%)
≥70	171 (57%)
*Education*	
Primary	124 (41.3%)
Secondary	112 (37.3%)
University	56 (18.7%)
M.S., Ph.D.	8 (2.7%)
*Job*	
Unemployed	4 (1.3%)
Civil servant	16 (5.3%)
Private employee	34 (11.3%)
Freelancer	6 (2%)
Household	44 (14.7%)
Pensioner	195 (65%)
Other	1 (0.3%)
*Residency*	
Attica	131 (43.7%)
County capital	94 (31.3%)
Small town	28 (9.3%)
Rural	47 (15.7%)

**Table 2:** Characteristics concerning the state of health of patients.

	*N* (%)
*Medication with anxiolytics*	
Yes	86 (28.7%)
No	214 (71.3%)
*Medication with antidepressants*	
Yes	14 (4.7%)
No	286 (95.3%)
*Other diseases*	
Yes	128 (42.7%)
No	172 (57.3%)
*Informed about the state of health*	
Very	90 (30%)
Enough	190 (63.3%)
A little	19 (6.3%)
Not at all	1 (0.3%)
*Years of having the problem*	
<1 year	40 (13.3%)
2–5	45 (15%)
6–10	104 (34.7%)
11–15	55 (18.3%)
>15	56 (18.7%)
*Any family member that suffers from a disease of the circulatory system*	
Yes	200 (66.7%)
No	100 (33.3%)
*Have you ever been hospitalized for the same reason?*	
Yes	246 (82%)
No	54 (18%)
*Frequency of hospitalization *	
1 per year	165 (55%)
2 per year	47 (15.7%)
3 per year	18 (6%)
>3 per year	38 (12.7%)
*Consider yourself anxious? *	
Yes	200 (66.7%)
No	100 (33.3%)
*Did you retire because of your cardiac problem?*	
Yes	21 (7%)
No	279 (93%)
*Are you absent from work because of your cardiac problem?*	
Yes	40 (13.3%)
No	260 (86.7%)
*Relations with medical staff*	
Very good	157 (52.3%)
Good	130 (43.3%)
Moderate	12 (4%)
Bad	0 (0%)
Very bad	1 (0.3%)
*Relations with nursing staff*	
Very good	176 (58.7%)
Good	109 (36.3%)
Moderate	13 (4.3%)
Bad	1 (0.3%)
Very bad	1 (0.3%)
	*Median (IQR)*
*Days of hospitalization*	5 (4–6)

**Table 3:** Measuring impact of heart failure on quality of life.

	Median (IQR)
Total score MINNESOTA (range 0–105)	46 (35–54)
Physical state (range 0–40)	22 (17–26)
Mental state (range 0–25)	6 (4–11)

**Table 4:** Associations between patients' characteristics and quality of life.

	Total median (IQR)	Physical state median (IQR)	Mental state median (IQR)
*Age*	*p* = 0.301	*p* = 0.167	**p** < 0.001
<60	53 (41–57)	20 (17–24)	11 (9–13)^*∗*^
60–69	40 (35–64)	20,5 (17–28)	6 (4–12)
>69	46 (39–54)	23 (18–25)	6 (5–10)
*Education*	**p** = 0.002	**p** = 0.003	**p** < 0.001
Primary	46 (35–53)	22 (17–27)	5 (4–8)
Secondary	54 (35–62)^*∗*^	25 (17–28)^*∗*^	10 (4–12)^*∗*^
University, M.S., Ph.D.	42,5 (32,5–52)	20 (16–23)	6 (4–11)
*Job*	**p** < 0.001	**p** = 0.007	**p** < 0.001
Civil/private employee	58,5 (51–66)^*∗*^	26 (20–28)^*∗∗*^	12 (11-12)^*∗*^
Householder	54 (35–54)	25 (17–25)	11 (4–11)
Pensioner	45 (34–51)	22 (17–26)	5 (4–8)
Other	36 (22–52)	17 (7–20)	4 (2–11)
*Residency*	**p** < 0.001	*p* = 0.096	**p** < 0.001
Attica	41 (35–49)	21 (17–26)	6 (3–8)
County capital	54 (35–66)^*∗*^	25 (17–28)	11 (4–12)^*∗*^
Small town/ rural	46 (31–55)	22 (15–27)	5 (5–9)

^*∗*^Statistically significant different score from other categories, after Bonferroni correction (multiple comparisons *p* < 0.05). ^*∗∗*^Statistically significant different score from category “other,” after Bonferroni correction (multiple comparisons *p* < 0.05).

**Table 5:** Associations between patients characteristics concerning the state of health of patients and quality of life.

	Total median (IQR)	Physical state median (IQR)	Mental state median (IQR)
*Medication with anxiolytics*	**p** < 0.001	**p** < 0.001	**p** < 0.001
Yes	54 (50–66)	25 (24–28)	11 (9–12)
No	43 (35–50)	21 (17–24)	5 (4–8)
*Medication with antidepressants*	**p** < 0.001	**p** < 0.001	**p** < 0.001
Yes	64.5 (55–70)	28 (25–32)	14 (10–17)
No	46 (35–54)	22 (17–26)	6 (4–11)
*Other diseases*	*p* = 0.792	**p** < 0.001	*p* = 0.130
Yes	45 (37–55.5)	24 (19.5–28)	6 (5–9)
No	46 (35–54)	21.5 (17–25)	6.5 (4–12)
*Informed about the state of health*	*p* = 0.224	*p* = 0.031	*p* = 0.542
Very	46 (31–66)	24 (17–28)	6 (3–12)
Enough	46 (35–54)	22 (17–25)	6 (4–11)
A little/not at all	48 (43–57)	25 (18–31)^*∗*^	10 (5–11)
*Years of having the problem*	**p** < 0.001	**p** < 0.001	**p** < 0.001
Less than two years	42.5 (28–53)	18.5 (14.5–30)	6 (3–8)
2–5	46 (36–54)	20 (17–22)	10 (5–12)
6–10	54 (41–64.5)^*∗*^	25 (17–28)^*∗*^	11 (4–12)^*∗*^
11–15	46 (39–46)	22 (21–23)	5 (5-6)
>15	35 (29.5–52.5)	20.5 (16–26)	4 (3–6)
*Any family member that suffers from a disease of the circulatory system*	*p* = 0.064	*p* = 0.603	**p** < 0.001
Yes	46 (35–54.5)	22 (17–25)	7.5 (5–11)
No	44 (32.5–54)	23 (16–28)	5.5 (3–8)
*Have you ever been hospitalized for the same reason?*	**p** = 0.005	**p** < 0.001	*p* = 0.702
Yes	46 (35–56)	23 (17–27)	6 (4–11)
No	41.5 (31–50)	18 (15–22)	6 (4–10)
*Frequency of hospitalization *	*p* = 0.795	**p** = 0.035	**p** = 0.015
1 per year	48 (35–54)	23 (17–26)	8 (4–12)
2 per year	46 (35–50)	22 (20–22)^*∗*^	5 (4–8)^*∗*^
3 per year	41.5 (35–57)	23.5 (19–29)	6 (4–9)
>3 per year	45.5 (37–56)	24.5 (17–30)	6.5 (3–8)
*Consider yourself anxious?*	**p** < 0.001	**p** < 0.001	**p** < 0.001
Yes	46 (36.5–56.5)	23 (17–27)	7 (5–11)
No	41 (31–51)	20 (15.5–24)	5 (3–8)
*Did you retire because of your cardiac problem?*	**p** < 0.001	**p** = 0.003	**p** = 0.003
Yes	33 (25–37)	17 (15–20)	4 (3–6)
No	46 (35–55)	22 (17–27)	6 (4–11)
*Are you absent from work because of your cardiac problem?*	**p** < 0.001	**p** = 0.023	**p** < 0.001
Yes	35.5 (25–51)	19 (11.5–26)	4.5 (3–9)
No	46 (35–54.5)	22 (17–26.5)	6 (4–11)
*Relations with medical staff*	*p* = 0.588	*p* = 0.889	**p** = 0.015
Very good	46 (36–55)	22 (17–28)	6 (5–11)
Good	45 (35–54)	23 (17–25)	6.5 (4–11)
Below moderate	48 (46–57)	21 (18–23)	11 (10–13)^*∗*^
*Relations with nursing staff*	*p* = 0.053	*p* = 0.321	**p** < 0.001
Very good	46 (37–54)	22.5 (18–26.5)	6 (5–11)
Good	41 (35–54)	20 (17–26)	5 (4–8)
Below moderate	52 (42–59)	22 (17–28)	11 (10–14)^*∗*^
	*Spearman's rho*	*Spearman's rho*	*Spearman's rho*
*Days of hospitalization*	0.039 (*p* = 0.497)	0.059 (*p* = 0.305)	0.038 (*p* = 0.513)

^*∗*^Statistically significant different score from other categories, after Bonferroni correction (multiple comparisons *p* < 0.05).

**Table 6:** Assessment of the effect of the factors on quality of life.

	Total *β* coef. (95% CI)	*p* value
*Job*		
Civil/private employee	Ref. Cat	
Householder	−17.6 (−23.15–−12.04)	**<0.001**
Pensioner	−4.63 (−10.75–1.5)	0.138
Other	−17.37 (−25.66–−9.08)	**<0.001**
*Medication with anxiolytics*		
Yes	Ref. Cat	
No	−5.93 (−9.82–−2.04)	**0.003**
*Medication with antidepressants*		
Yes	Ref. Cat	
No	−15.58 (−22.5–−8.66)	**<0.001**
*Have you ever been hospitalized for the same reason?*		
Yes	Ref. Cat	
No	−7.04 (−11.37–−2.71)	**0.002**
*Did you retire because of your cardiac problem?*		
Yes	Ref. Cat	
No	7.02 (0.53–13.51)	**0.034**

**Table 7:** Assessment of the effect of the factors on quality of life (subscales).

	Physical State *β* coef. (95% CI)	*p* value	Mental state *β* coef. (95% CI)	*p* value
*Age*				
<60	—		Ref. Cat	
60–69	—		−1.91 (−3.57–−0.26)	**0.024**
>69	—		0.26 (−1.54–2.07)	0.776
*Education*				
Primary	Ref. Cat		Ref. Cat	
Secondary	1.86 (−0.54–4.26)	0.129	1.22 (0.04–2.4)	**0.042**
University, M.S., Ph.D.	0.01 (−2.37–2.4)	0.990	0.14 (−0.97–1.24)	0.810
*Job*				
Civil/private employee	Ref. Cat		Ref. Cat	
Householder	−6.43 (−9.49–−3.37)	**<0.001**	−6.06 (−7.71–−4.41)	**<0.001**
Pensioner	−4.74 (−7.63–−1.84)	**0.001**	−3.06 (−4.94–−1.18)	**0.002**
Other	−9.27 (−13.41–−5.12)	**<0.001**	−4.73 (−6.83–−2.63)	**<0.001**
*Residency*				
Attica	—		Ref. Cat	
County capital	—		2.15 (0.57–3.74)	**0.008**
Small town/rural	—		0.25 (−0.73–1.24)	0.615
*Medication with anxiolytics*				
Yes	Ref. Cat		Ref. Cat	
No	−2.86 (−4.84–−0.89)	**0.005**	−1.44 (−2.45–−0.44)	**0.005**
*Medication with antidepressants*				
Yes	Ref. Cat		Ref. Cat	
No	−2.8 (−6.58–0.98)	0.146	−5.28 (−7.13–−3.43)	**<0.001**
*Other diseases*				
Yes	Ref. Cat		—	
No	−3.61 (−5.51–−1.71)	**<0.001**	—	
*Informed about the state of health*				
Very	Ref. Cat		—	
Enough	2.38 (0.55–4.22)	**0.011**	—	
A little/not at all	8.72 (5.37–12.06)	**<0.001**	—	
*Years of having the problem*				
<1	Ref. Cat		Ref. Cat	
2–5	0.06 (−3.45–3.57)	0.972	2.34 (0.61–4.07)	**0.008**
6–10	4.43 (1.05–7.8)	**0.010**	2.69 (1.03–4.35)	**0.002**
11–15	5.06 (1.53–8.59)	**0.005**	0.97 (−0.76–2.7)	0.270
>15	−0.25 (−3.69–3.19)	0.888	1.15 (−0.54–2.84)	0.180
*Have you ever been hospitalized for the same reason?*				
Yes	Ref. Cat		—	
No	−4.9 (−7.81–−1.98)	**0.001**	—	
*Frequency of hospitalization *				
1 per year	Ref. Cat		Ref. Cat	
2 per year	1.85 (−0.67–4.37)	0.149	0.49 (−0.77–1.74)	0.446
3 per year	3.16 (−0.09–6.4)	0.056	0.18 (−1.42–1.77)	0.828
>3 per year	6.16 (3.59–8.72)	**<0.001**	1.72 (0.52–2.93)	**0.005**
*Consider yourself anxious? *				
Yes	Ref. Cat		Ref. Cat	
No	0.63 (−1.21–2.47)	0.501	−1.15 (−2.15–−0.16)	**0.023**
*Did you retire because of your cardiac problem?*				
Yes	Ref. Cat		Ref. Cat	
No	3.8 (0.51–7.1)	**0.024**	−0.61 (−2.3–1.08)	0.479
*Relations with medical staff*				
Very good	—		Ref. Cat	
Good	—		1.79 (0.38–3.2)	**0.013**
Below moderate	—		5.01 (2.46–7.55)	**<0.001**
